# Genome-resolved insights into the bacterial phylum WOR-3: hydrogenotrophic metabolism and unique carbon fixation via archaeal form III RuBisCO

**DOI:** 10.1128/msystems.01178-25

**Published:** 2025-10-02

**Authors:** Jianxiong Zeng, Wenzhe Hu, Licao Chang, Zhengshuang Hua, Geng Wu, Yun Fang, Guowei Wang, Chunqiao Xiao, Jun Liu

**Affiliations:** 1 Key Laboratory for Green Chemical Process of Ministry of Education, School of Environmental Ecology and Biological Engineering, Wuhan Institute of Technology 34756, Wuhan, People's Republic of China; 2 State Key Laboratory of Agricultural Microbiology, State Environmental Protection Key Laboratory of Soil Health and Green Remediation, College of Resources and Environment, Huazhong Agricultural University 47895, Wuhan, People's Republic of China; 3 Department of Environmental Science and Engineering, State Key Laboratory of Advanced Environmental Technology, University of Science and Technology of China 12652, Hefei, People's Republic of China; 4 State Key Laboratory of Biogeology and Environmental Geology, China University of Geosciences 12564, Wuhan, People's Republic of China; Los Alamos National Laboratory, Los Alamos, New Mexico, USA

**Keywords:** WOR-3, metabolism reconstruction, biogeochemical cycle, RuBisCO, Rnf complex

## Abstract

The WOR-3 phylum is widely distributed in various environments, including hot springs, marine ecosystems, and hydrothermal vents, yet its ecological roles and metabolic capabilities remain poorly understood. In this study, we analyzed 181 medium- to high-quality metagenome-assembled genomes, including 59 newly reconstructed from environmental samples and 122 retrieved from public databases. Phylogenetic analyses resolved the WOR-3 lineage into four subgroups (subgroup 1–4). Metabolic reconstruction revealed significant divergence of the carbon, sulfur, nitrogen, and hydrogen metabolism pathways among the different subgroups. Subgroup 1 was characterized by fermentative metabolism involving formate and ethanol and uniquely exhibited potential for carbon fixation via the Calvin cycle, as indicated by the presence of ribulose-1,5-bisphosphate carboxylase/oxygenase (RuBisCO) gene. Notably, WOR-3 RuBisCO is phylogenetically affiliated with archaeal form III, although the carbon fixation pathway follows the canonical bacterial Calvin cycle—a feature of potential evolutionary significance. Subgroup 3 exhibits metabolic versatility, including genes for dissimilatory sulfate reduction, sulfur oxidation, partial denitrification, and fatty acid degradation. In addition, all subgroups harbored key components of hydrogen metabolism, including widespread NiFe hydrogenases, supporting H_2_-dependent electron transfer and energy conservation. Within the WOR-3 lineage, the coexistence of two respiratory enzyme systems—the Rnf complex and the oxidative phosphorylation respiratory chain—indicates distinct anaerobic and aerobic metabolic lifestyles, respectively. Collectively, this study expands the genomic framework for the WOR-3 phylum and provides novel insights into the metabolic versatility and ecological functions of this previously uncharacterized lineage in biogeochemical cycles of carbon, nitrogen, and sulfur.

**IMPORTANCE:**

The WOR-3 phylum represents a widespread but poorly understood bacterial lineage inhabiting diverse various environments. By integrating 181 metagenome-assembled genomes, including 59 newly reconstructed, this study provides the most comprehensive genomic framework to date for WOR-3. Phylogenomic and metabolic reconstruction revealed four distinct subgroups with divergent capacities for carbon, sulfur, and nitrogen metabolism. Notably, subgroup 1 encodes a complete Calvin-Benson-Bassham cycle featuring an archaeal-type form III ribulose-1,5-bisphosphate carboxylase/oxygenase, suggesting an unusual evolutionary trajectory for carbon fixation in this lineage. Subgroup 3 exhibits versatile metabolic potential, including dissimilatory sulfur metabolism, partial denitrification, and fatty acid degradation, highlighting its possible roles in multiple biogeochemical processes. These findings not only expand the taxonomic and functional landscape of the WOR-3 phylum but also offer key insights into its ecological roles in global element cycling.

## INTRODUCTION

Despite advances in cultivation techniques, only a minor fraction of microorganisms in natural environments can be cultured, with most lineages remaining poorly characterized. The bacterial phylum WOR-3, initially discovered in hot spring environments, exemplifies this poorly characterized microorganism. As research advanced, the ecological adaptations of this phylum garnered increased attention. The earliest report of WOR-3 dates back to 1994, when a 16S rRNA gene sequence was recovered from Octopus Spring in Yellowstone National Park and tentatively classified as “*Candidatus* Hydrothermota” (1). In order to explore the role of WOR-3 in biogeochemical cycles, researchers have investigated its metabolic capacity. The advent of high-throughput sequencing technology has rendered metagenomic analysis an indispensable instrument in the analysis of microorganisms. In 2016, metagenome-assembled genomes (MAGs) designated T1.2 were recovered from filamentous microbial mat samples collected at Bechler Spring in Yellowstone National Park. The study proposed assigning T1.2 to a candidate phylum-level lineage named “Pyropristinus” and referred to the specific organism represented by T1.2 as “*Candidatus* Caldipriscus” (2). A 2017 publication in the field of estuarine sediments reported that the genome of the metagenome spliced WOR-3. The authors of the study suggested that this organism should be designated “*Candidatus* Stahlbacteria” (3). However, the assembled WOR-3 genome in this literature was less than 60% complete, resulting in a failure to fully reveal its metabolic potential. Cultivation of WOR-3 members has rarely been reported. As of March 2025, the first representative pure culture from a WOR-3 lineage—designated strain sy37—was successfully isolated from deep-sea hydrothermal fluids. Phylogenomic analysis classified this strain within the class Hydrothermia (4).

Reports on the metabolism of WOR-3 are complex and diverse, indicating that the WOR-3 lineage is a metabolically versatile phylum. Metabolic reconstruction of the “*Candidatus* Caldipriscus” population in a previous study suggests it is an obligate chemoorganoheterotroph (2). Metabolic reconstruction revealed that the genomes of “*Candidatus* Caldipriscus,” the pure culture strain sy37, and WOR-3 genomes recovered from environments such as the Great Boiling Spring and anaerobic reactors (S04_bin.1_UBA1063, S04_bin.2_UBA1063) possess metabolic capabilities for glycolysis and gluconeogenesis (4–6). The presence of the tricarboxylic acid (TCA) cycle remains controversial, as a complete set of TCA cycle enzymes could not be annotated in S04_bin.1_UBA1063 and S04_bin.2_UBA1063 (7). The presence of glycolysis and gluconeogenesis suggests that WOR-3 can utilize high-quality carbon sources such as glucose, but it also appears capable of utilizing small organic molecules, for example, acetate metabolism, ethanol fermentation, and even certain amino acids (serine and alanine) (6, 8). Furthermore, hydrogenases ([FeFe] hydrogenases of group A) were successfully annotated in “*Candidatus* Stahlbacteria” and S04_bin.2_UBA1063 within the WOR-3 lineage. A study on a WOR-3 genome recovered from an anaerobic reactor used metatranscriptomic analysis to reveal the expression of hydrogen production-related enzymes in the reconstructed WOR-3 genome, while showing no expression of quinone- and cytochrome c-dependent oxidative respiratory enzymes (7). This provides strong evidence that WOR-3 is capable of hydrogen metabolism (3, 6, 7). WOR-3 is also capable of fatty acid degradation (β-oxidation pathway), as demonstrated in “*Candidatus* Stahlbacteria.” Analysis of WOR-3 genomes from public databases through extensive metabolic reconstruction identified key processes in sulfur and nitrogen metabolism. Nitrogen metabolism includes nitrite oxidation, dissimilatory nitrate reduction (DNR), nitrite reduction, and nitrous oxide reduction, while sulfur metabolism encompasses dissimilatory sulfate reduction, sulfhydrogenase activity, and sulfide oxidation (4). Meanwhile, growth tests demonstrated the ability to utilize elemental sulfur. Regarding energy conservation, components of the oxidative respiratory chain were found in both “*Candidatus* Caldipriscus” and strain sy37, while S04_bin.1_UBA1063 and S04_bin.2_UBA1063 from anaerobic reactor environments suggest WOR-3 may employ different respiratory strategies. Autotrophic microorganisms hold a special position as primary producers in microbial communities, making carbon fixation an important metabolic function of interest; however, extensive research has seemingly paid little attention to the carbon fixation capabilities of WOR-3. Although the isolation of the pure culture strain sy37 provided crucial evidence for exploring WOR-3’s metabolic functions and enabled significant metabolic reconstruction of existing WOR-3 lineages, strain sy37 conclusions primarily focused on the physiological characteristics, overlooking the metabolic differences exhibited across the entire WOR-3 lineage.

Given the very limited number of high-quality genomes of WOR-3 that are currently available, its ecological functional diversity, habitat distribution, and environmental adaptations remain to be explored. To address these issues, we retrieved 57 genomes from sediments (56 from hot spring and 1 from lake), 2 genomes from an anaerobic bioreactor, and 122 genomes from published genomes. By integrating existing databases with newly sampled and assembled WOR-3 genome data, this work presents an integrated and comprehensive analysis of the phylogeny, global habitat distribution, and metabolic potential of WOR-3. Based on our findings, the WOR-3 phylum diverges into four phylogenetically distinct subgroups, each exhibiting unique metabolic features. This delineation provides a functional classification framework for WOR-3, highlighting its remarkable metabolic versatility.

## MATERIALS AND METHODS

### Sampling, DNA extraction, and sequencing

The sampling expedition was conducted from January 2021 to August 2023. Surface sediment samples were collected from the Tengchong and Quzhuomu hot springs, China. These sediments were collected into 50 mL sterile centrifuge tubes and immediately kept on dry ice until they reached the laboratory. Genomic DNA was extracted from each 10 g sediment sample using a modified phenol-chloroform method (9). A standard shotgun library with an insert size of 300 bp was inserted and then sequenced on the Illumina Novaseq platform (paired-end 150 bp mode).

### Metagenomic analysis

Raw reads were pretreated using a custom Perl script and Sickle as previously reported (10). Then the resultant high-quality reads for each sample were assembled independently using SPAdes (version 3.15.2) with the parameters “-k 33,55,77,99 --meta.” The scaffolds were binned based on the tetranucleotide frequencies and scaffold coverage using MetaBAT (version 2.12.1) (11) with the parameters “-m 2000 --unbinned.” The preliminary classification of all bins was confirmed using the Genome Taxonomy Database Toolkit (GTDB-Tk) (12), and genome bins belonging to the WOR-3 phylum were selected. The retrieved genomes were de-duplicated using dRep software (13), and low-quality MAGs (completeness < 50% and contamination < 10%) were removed. The final data set comprised 181 medium- to high-quality MAGs, classified as follows: medium-quality MAGs (completeness ≥ 50%, contamination <10%) and high-quality MAGs (completeness > 90%, contamination < 5%). These included 59 genomes assembled from our samples, 121 genomes sourced from public databases, and 1 genome obtained via whole-genome sequencing (14). As described previously (10), they were re-assembled using the recruited reads through BBMap (15) and were examined manually to remove possible contamination. Their completeness, contamination, and strain heterogeneity were evaluated by using CheckM (16). These curated genomes were used for the subsequent analyses including functional annotation, phylogenomic and phylogenetic analyses, and metabolic inference. The average amino acid identity (AAI) and average nucleotide identity (OrthoANI) of these 181 MAGs were then calculated using EzAAI (17) and OrthoANI (18), respectively.

### Genome annotation and metabolic reconstruction

Gene calling of MAGs was performed using Prodigal (v.2.6.3) software in “-p single” mode (19). The protein-coding genes were annotated based on comparisons with the NCBI-nr, KEGG (20), EggNOG (21), and Pfam databases using DIAMOND with E-value ≤1e^−5^ (22). In consideration of the annotation results outlined above, a systematic reconstruction of the metabolic pathways of each genome was conducted, with a particular emphasis on pivotal metabolic pathways such as carbon metabolism (including carbon fixation), sulfur metabolism, nitrogen metabolism, energy conservation, and hydrogen metabolism. In this study, we applied metabolic inference criteria to enhance the reliability of functional predictions. Pathway presence was inferred only when multiple diagnostic genes were co-annotated, avoiding reliance on single markers. For multi-subunit complexes, presence was assigned only if more than 50% of the core subunits were identified. This conservative approach minimizes false-positive annotations and ensures a robust assessment of metabolic potential. Metabolic potential was determined through manual curation of pathway presence/absence based on diagnostic gene detection, generating a binary matrix representing functional capabilities across all analyzed genomes (23).

### Phylogenomic and phylogenetic analysis

Phylogenomic analysis was performed on the 181 representative MAGs in this study. One exogenous genome from the *Gemmatimonadota* phylum was additionally incorporated for comparative analysis. Briefly, a concatenated multiple sequence alignment of 120 conserved bacterial marker genes was retrieved using GTDB-Tk. Poorly aligned regions were trimmed with trimAL under parameters “-gt 0.95 -cons 50.” A phylogenomic tree was constructed based on a concatenation of 120 bacterial marker proteins using IQ-TREE with the parameters “LG+F+L+L+R9 -alrt 1000 -bb 1000” (24).

16S rRNA sequences from representative MAGs of WOR-3 lineage were predicted using the ssu_finder module of CheckM (16). Sequences shorter than 800 bp were discarded, retaining only the longest 16S rRNA sequence per MAG when multiple copies were present. Moreover, 16S rRNA gene sequences from WOR-3 genomes and environmental 16S rRNA gene sequences were aligned using SINA (25), and then the alignment was filtered by trimAL with parameters set to “-gt 0.95 -cons 50” (26). 16S rRNA gene-based phylogenetic tree was constructed using IQ-TREE with the parameters “SYM+I+I+R6.”

The ribulose-1,5-bisphosphate carboxylase/oxygenase (RuBisCO) enzyme—a key component of the Calvin-Benson-Bassham (CBB) cycle—exhibits substantial diversity in its large subunit (*rbcL*). For phylogenetic analysis, 137 *rbc*L protein sequences were retrieved from public NCBI databases, supplemented with five novel *rbc*L sequences from WOR-3 lineages. To determine the lifestyle preference of the Rnf complex in WOR-3, protein sequences of the Rnf C subunit were separately collected from anaerobic, aerobic, or facultative anaerobic organisms. Multiple sequence alignment was performed using MAFFT, followed by trimming of poorly aligned regions with trimAL under identical parameters (“-gt 0.95 -cons 50”) as previously applied (27). Furthermore, the phylogenetic tree of *rbc*L subunit and *rnf*C sequences was constructed using IQ-TREE with the parameters “LG+R6” and “LG+I+I+R6,” respectively. The constructed phylogenetic trees were then visualized using iTOL to display and interpret the analysis results more clearly (28). To assess the distribution patterns of the Rnf complex versus cytochrome oxidase across MAGs, a clustered heatmap was generated in R using the “pheatmap” package (29), visualizing the binary presence/absence matrix of associated genes.

## RESULTS AND DISCUSSION

### Genomic diversity and biogeography of WOR-3

**Fig 1:**
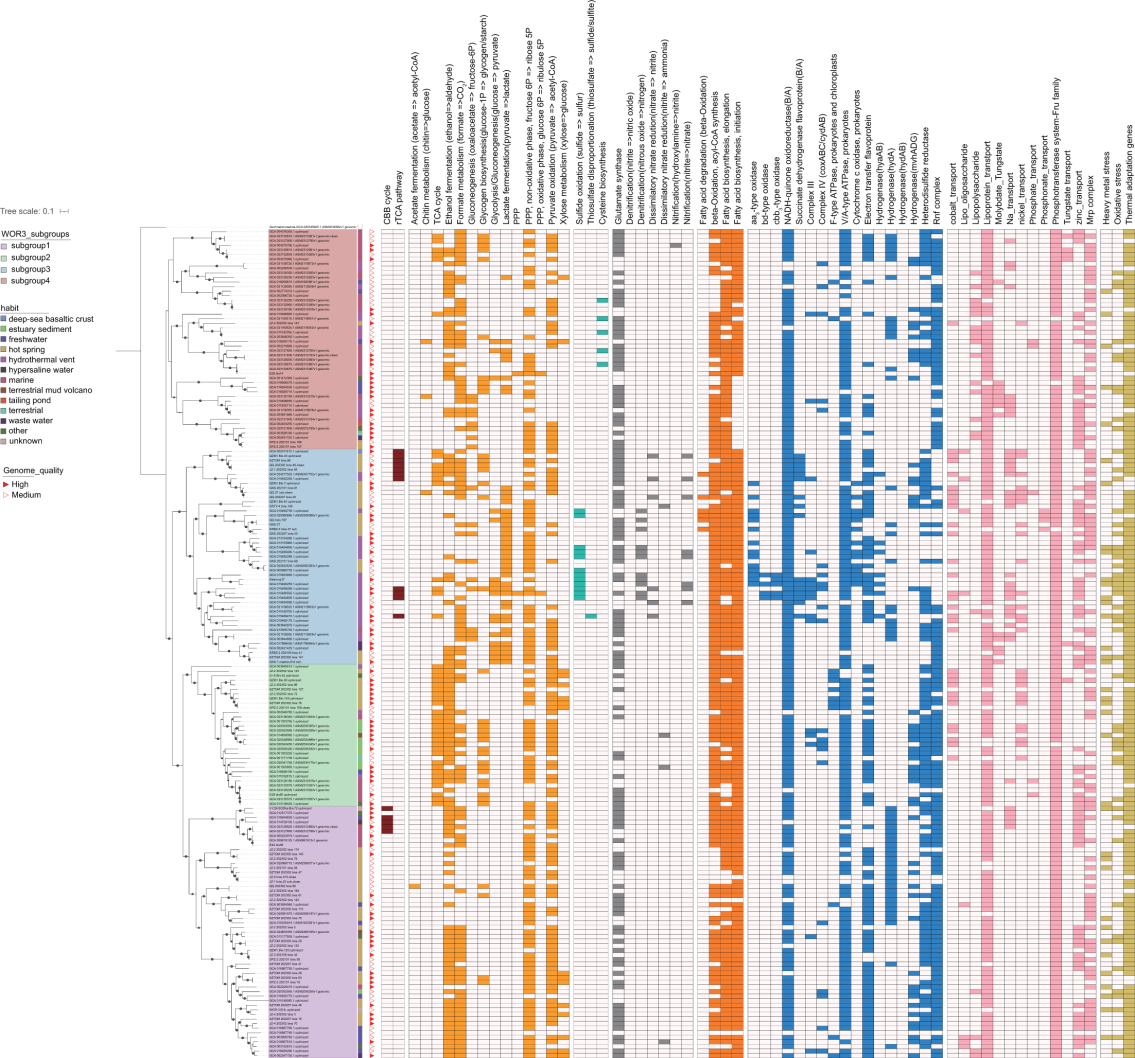
Metabolic-functional mapping, phylogenomic tree, and habitats of WOR-3. The phylogenomic tree of WOR-3 constructed based on 120 conserved marker proteins. Bootstrap values for phylogenetic trees greater than 75 are marked with gray solid black-edged circles. The genomes of *Gemmatimonadota* were used as exogenous genome. Metabolic function presence status is marked with rectangular squares of different colors and absent metabolic functions are white rectangular squares. The metabolic function mapping includes a total of nine functional categories: carbon metabolism, carbon fixation, sulfur metabolism, nitrogen metabolism, electron transport and hydrogen metabolism, fatty acid metabolism, transporter proteins, and stress adaptation. PPP, pentose phosphate pathway.

**Fig 2:**
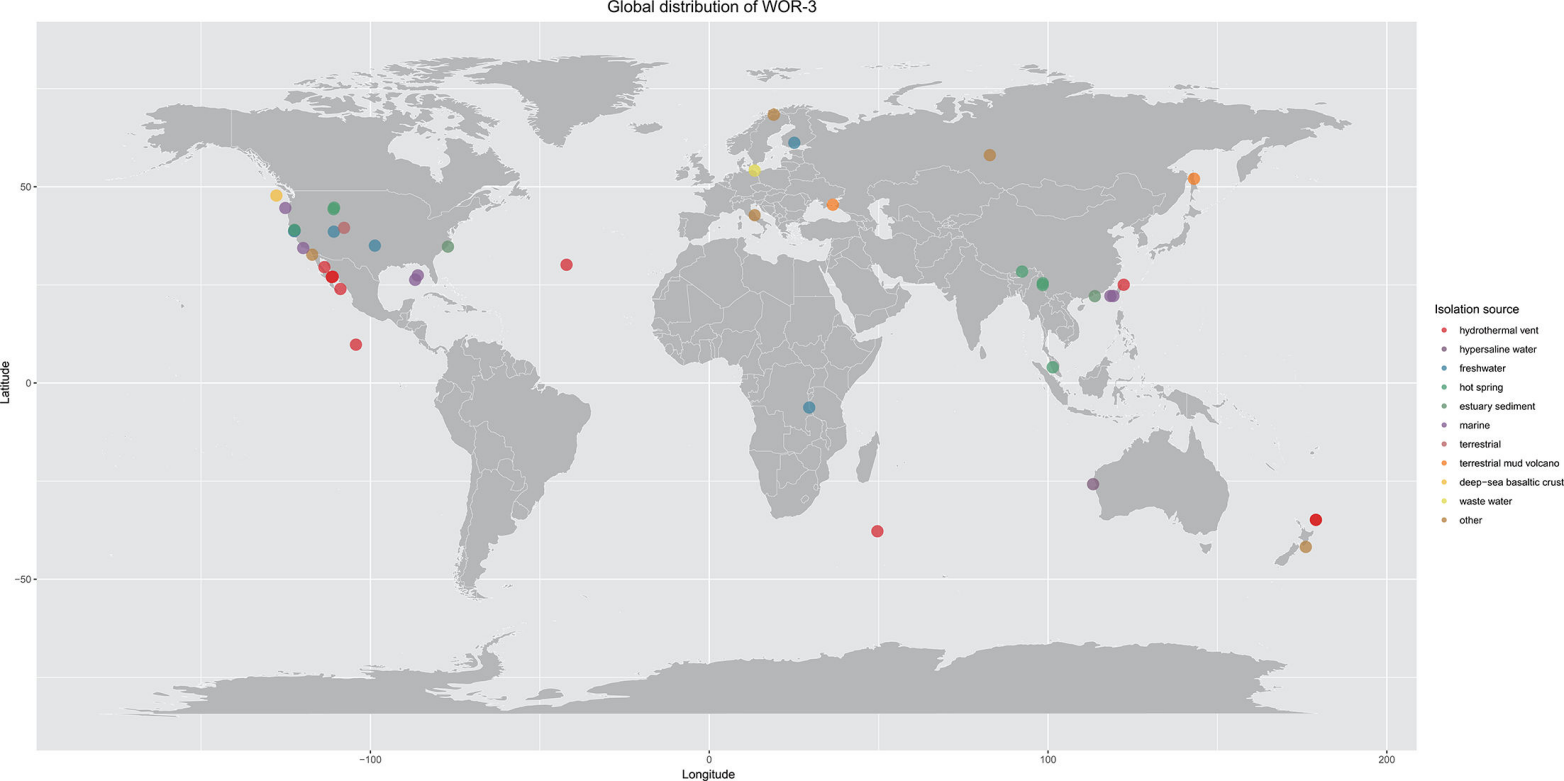
The global distribution map of WOR-3. Solid circles indicate the global distribution of MAGs, different colored circles represent different habitats of WOR-3.

To decipher the physiology of WOR-3, the 59 MAGs retrieved in this study and another 122 MAGs published previously were analyzed (Table S1). The lengths of these genomes ranged from 0.83 Mb to 4.19 Mb, the completeness ranged from 50.59% to 97.8%, and the contamination levels were observed to be less than 7.33%. Phylogenomic analysis revealed that the 181 genomes were classified into four subgroups, which was supported by the GTDB analysis and ANI/AAI analyses (Fig. 1; Fig. S1; Table S2). The number of each subgroup is as follows: subgroup 1 (*n*_MAGs_ = 55, *n*_samples_ = 29), subgroup 2 (*n*_MAGs_ = 31, *n*_samples_ = 9), subgroup 3 (*n*_MAGs_ = 47, *n*_samples_ = 18), subgroup 4 (*n*_MAGs_ = 48, *n*_samples_ = 3). In order to comprehensively understand the ecological distribution characteristics of WOR-3, this study traced the environmental origin of WOR-3 based on the 16S rRNA gene sequences (Table S3) and the information of WOR-3 MAGs available in the GTDB database. It is difficult to obtain the complete 16S rRNA gene because of the incompleteness of MAGs. Some sequences in the 16S rRNA gene phylogenetic tree (Fig. S2) are inconsistent with the taxonomic assignments from GTDB and the inferred subgroup classification. Two sequences classified as belonging to class c_UBA3073 in GTDB were found to cluster phylogenetically closer to class Hydrothermia in the 16S rRNA tree. This discrepancy may result from limited phylogenetic resolution due to the use of a single marker gene or could be influenced by lower genome completeness. The results showed that since the discovery of WOR-3 from the hot spring environment of Yellowstone Park, USA, in 1994, more than 39 samples of WOR-3 were successively discovered in different environments from 2004 to 2011 by 16S rRNA gene sequence analysis. The application of high-throughput sequencing technology has facilitated the successful identification of metagenomic sequences of WOR-3 through the assembly of DNA fragments from environmental samples. Since 2015, the assembly of the WOR-3 genome has been achieved, leading to a substantial increase in the number of MAGs of WOR-3. The habitats of WOR-3 are highly diverse, with a primary presence in aquatic environments, including hot springs (*n* = 62), hydrothermal vents (*n* = 37), marine environments (*n* = 35), freshwater sources (*n* = 17), wastewater (*n* = 4), and hypersaline water environments (*n* = 4). These environments are not exclusive, however, as WOR-3 has also been identified in terrestrial habitats, including estuarine sediment (estuary sediment, *n* = 10), terrestrial mud volcanoes (terrestrial mud volcano, *n* = 2), oil reservoirs (oil reservoir, *n* = 2), and bioreactors (bioreactor, *n* = 2) (Fig. 2; Table S1). Each of the four explored subgroups of WOR-3 has been found to exist in both aquatic (e.g., hydrothermal vents, hot springs, oceans, etc.) and terrestrial environments (e.g., crustal, terrestrial mud volcanoes, etc.), but the vast majority of the genomes are predominantly distributed in aquatic environments. In terms of latitudinal distribution, WOR-3 is mainly concentrated in the middle and low latitudes (41.77°S–68.35°N).

The extensive environmental distribution indicates the metabolic diversity of WOR-3 and its strong adaptive capacity of these little-known microorganisms to adapt to both normal and harsh environments (Fig. 2). Its tolerance to temperature is impressive, such as 67°C volcanic hot springs in southwest Iceland (30) and 75°C–93°C hot springs in Yellowstone National Park (2, 31, 32). WOR-3 was similarly abundant in the hot spring (>50°C), with the relative abundance of ~0.1% (7). The abundance of WOR-3 exhibited substantial variation across diverse reservoir environments, with significantly increased levels observed in sulfate-rich oil degradation environments (4.19%–6.05%) (6).

### Carbon metabolism

The central carbon metabolism pathway, including glycolysis/gluconeogenesis, the pentose phosphate pathway, and the TCA cycle, provides the carbon skeleton for WOR-3 and generates ATP and NADH through substrate-level phosphorylation (33). It is widely reported that the WOR-3 lineage is capable of carbon metabolism. Metabolic reconstruction of “*Candidatus* Caldipriscus” (recovered from Yellowstone National Park) revealed the presence of complete glycolysis genes, various polysaccharide hydrolases, and genes involved in fatty acid degradation (2). However, it lacks the capacity for anaerobic fermentation, evidenced by the absence of key enzymes including alcohol dehydrogenase, acetate kinase, and formate dehydrogenase (FDH). In contrast, our functional assessment of “*Candidatus* Caldipriscus” diverges from previous reports, specifically regarding deficiencies in glycolysis and TCA cycle. The accession number for “*Candidatus* Caldipructus” in the public database is “GCA_020832835.1.” We determined functional deficiencies based on the absence of two key enzymes: 6-phosphofructokinase (involved in glycolysis) and isocitrate dehydrogenase (IDH) (involved in the TCA cycle). This indicates that “*Candidatus* Caldipriscus” lacks critical steps in both glycolysis and the TCA cycle, which are essential for metabolic processes (Table S4) (2). The pure-culture strain sy37 exhibits broad oxidative metabolism of carbohydrate substrates but lacks carbon fixation capabilities (4).

**Fig 3:**
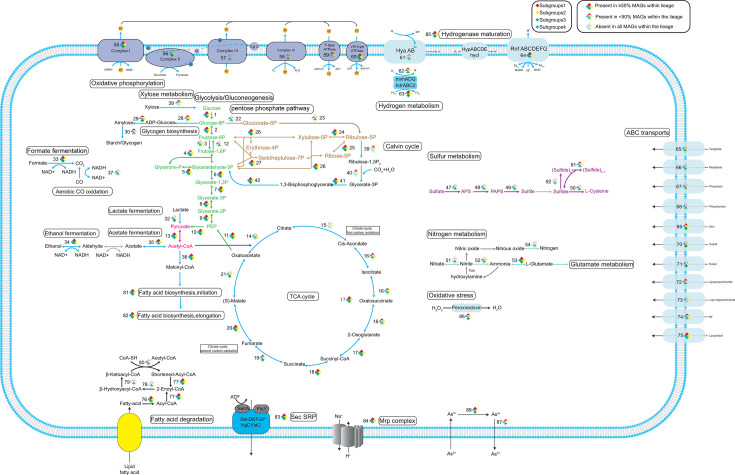
Metabolic network diagram for WOR-3. The four colored diamonds indicate different subgroups. The prisms have three states, solid diamonds indicate that the corresponding genes are present in ≥50% of the MAGs of the subgroup, triangles indicate <50% and >0%, and hollow prisms indicate that the corresponding genes are missing from all MAGs of the subgroup. For full names and copy numbers of the genes, see Table S4.

It is argued that the glycolysis/gluconeogenesis pathways and TCA cycle of WOR-3 are more complete and are capable of obtaining energy through substrate-level phosphorylation. The glycolysis pathway is subject to three rate-limiting enzymes: glucokinase (*glk*), 6-phosphofructokinase (*pfk*), and pyruvate/orthophosphate dikinase (*ppdk*) (23). The presence of dikinase (*ppd*K) is an important marker in determining the presence of this metabolic pathway. In the present study, glucokinase and pyruvate/orthophosphate dikinase were annotated in almost all genomes (Fig. 3; Table S4). However, the deletion of 6-phosphofructokinase in subgroup 4 resulted in only one genome in this group with intact glycolytic capacity, a phenomenon that is similar to that observed in the thermophilic archaeon “*Candidatus* Caldiarchaeum subterraneum” (34).

The gluconeogenesis pathway shares a number of enzymes with the glycolysis pathway, and some of the enzymes involved in irreversible reactions (phosphoenolpyruvate carboxykinase [*pck*A], fructose-1,6-bisphosphatase [*FBP*], and glyceraldehyde 3-phosphate dehydrogenase [*GAPDH*]) are key enzymes in this pathway (35). It was further demonstrated that phosphoenolpyruvate carboxykinase was classified as both GTP-dependent and ATP-dependent. The analysis revealed that these enzymes evidenced a distinct preference for different subgroups, with the GTP-dependent enzyme predominantly found in subgroup 2 and the ATP-dependent enzyme predominantly distributed in subgroups 1 and 3. The study concluded with the observation that only one genome in subgroup 4 contained both enzymes. Despite the fact that the majority of glycolysis/gluconeogenesis-related enzymes had been annotated in WOR-3, enzymes involved in the conversion of fructose 1,6-diphosphate to fructose 1-phosphate were found in low numbers in subgroup 1. The finding that fructose 1,6-bisphosphatase was annotated in only 15 genomes across all subgroups suggests that WOR-3 does not possess a complete gluconeogenesis function (Fig. 3; Table S4).

The rate-limiting enzymes of the TCA cycle include citrate synthase (*cs*), IDH, and 2-oxoglutarate ferredoxin oxidoreductase (*kor*ABCD). Applying these three enzymes as criteria for determination, the complete TCA cycle was annotated in subgroup 2 (*n* = 24), subgroup 3 (*n* = 11), and subgroup 4 (*n* = 9) (Table S2). This study found that combining the results of the annotation of glycolysis/gluconeogenesis and TCA cycle, the four subgroups of WOR-3 showed significant differences in the central metabolic pathways. It is clear that subgroup 1 lacks the ability of glycolysis/gluconeogenesis and TCA cycle, suggesting that it is unable to harness glucose as a carbon source, whereas subgroup 2 and subgroup 4 may have the ability of intact glycolysis/gluconeogenesis and TCA cycle; and subgroup 3 only lacks the function of gluconeogenesis, suggesting that it is able to leverage glucose for metabolism.

In the absence of the glycolysis pathway, it is hypothesized that subgroup 1 may rely on other carbon sources for metabolism. The presence of ethanol dehydrogenase (*adh*) in WOR-3 was identified, with the ability to oxidize alcohols to acetaldehyde, while generating the reducing power NADH and protecting cells from ethanol toxicity. This metabolic ability is prevalent in WOR-3, especially in subgroup 1, where 40 MAGs possess this pathway. WOR-3 has also been observed to possess the capability of converting formic acid to CO_2_ via FDH. Additionally, 33 genomes in subgroup 4 have been found to contain formate dehydrogenase with coenzyme F420 as the electron acceptor. Although the small and medium subunits of carbon monoxide (CO) dehydrogenase are annotated in WOR-3, the large subunit that performs the function is not annotated, suggesting that it may not be able to fully catalyze the conversion of CO and H_2_ to CO_2_ and protons. Interestingly, 16 genomes from subgroups 1 and 2 possess xylose isomerase, which is capable of converting xylose to glucose and entering the glycolysis pathway. Among them, 11 out of 16 were derived from our study, greatly expanding the number of xylose utilization members of WOR-3 (Fig. 3; Table S4). These MAGs were concentrated on the genealogical branch of the phylogenetic tree (Fig. 1).

Additionally, the pentose phosphate pathway (PPP) consisting of an oxidative phase and a non-oxidative phase was analyzed. The oxidative phase has been observed to be absent in many aerobic and thermophilic organisms (e.g., *Methanocaldococcus jannaschii*), whereas the non-oxidative phase has been identified as being ubiquitous in a wide range of organisms and is primarily responsible for providing RNA backbone precursors (36, 37). Key enzymes of the oxidative phase of the PPP, glucose-6-phosphate 1-dehydrogenase (*zwf*), 6-phosphogluconolactonase (*pgl*), and 6-phosphogluconate dehydrogenase (*gnd*) (23), were found in only a very small number of genomes, specifically those affiliated with subgroups 3 and 4. In addition, it was found that key genes of the non-oxidative phase were present in most genomes (*n* = 125) (Fig. 3; Table S4). Combined with the broad distribution of WOR-3 in hot spring environments, it can be deduced that although WOR-3 is devoid of the capability to produce NADH via the oxidative phase of the PPP, it relies on transketolase and transaldolase as a connection to the glycolysis/gluconeogenesis pathway and continues to provide intermediary cellular metabolism such as RNA backbone metabolites.

Previous studies have reported that WOR-3 may possess the potential for fatty acid degradation (3). However, current findings indicate that this metabolic capability does not appear to be universally present in WOR-3. In WOR-3, acyl-CoA synthetase—which activates fatty acids by forming acyl-CoA during beta-oxidation—is widely distributed across all genomes. The remaining enzymes (acyl-CoA dehydrogenase, enoyl-CoA hydratase, 3-hydroxyacyl-CoA dehydrogenase, and acetyl-CoA acyltransferase) are responsible for sequentially breaking down long-chain acyl-CoA into shortened intermediates, continuously generating acetyl-CoA along with reducing power (NADH and FADH₂) (38). Notably, only six genomes within subgroup 3 encode a complete enzyme set for long-chain acyl-CoA degradation, demonstrating this subgroup’s unique capacity for lipid-derived carbon metabolism through fatty acid β-oxidation within the WOR-3 phylum.

### Carbon fixation

**Fig 4:**
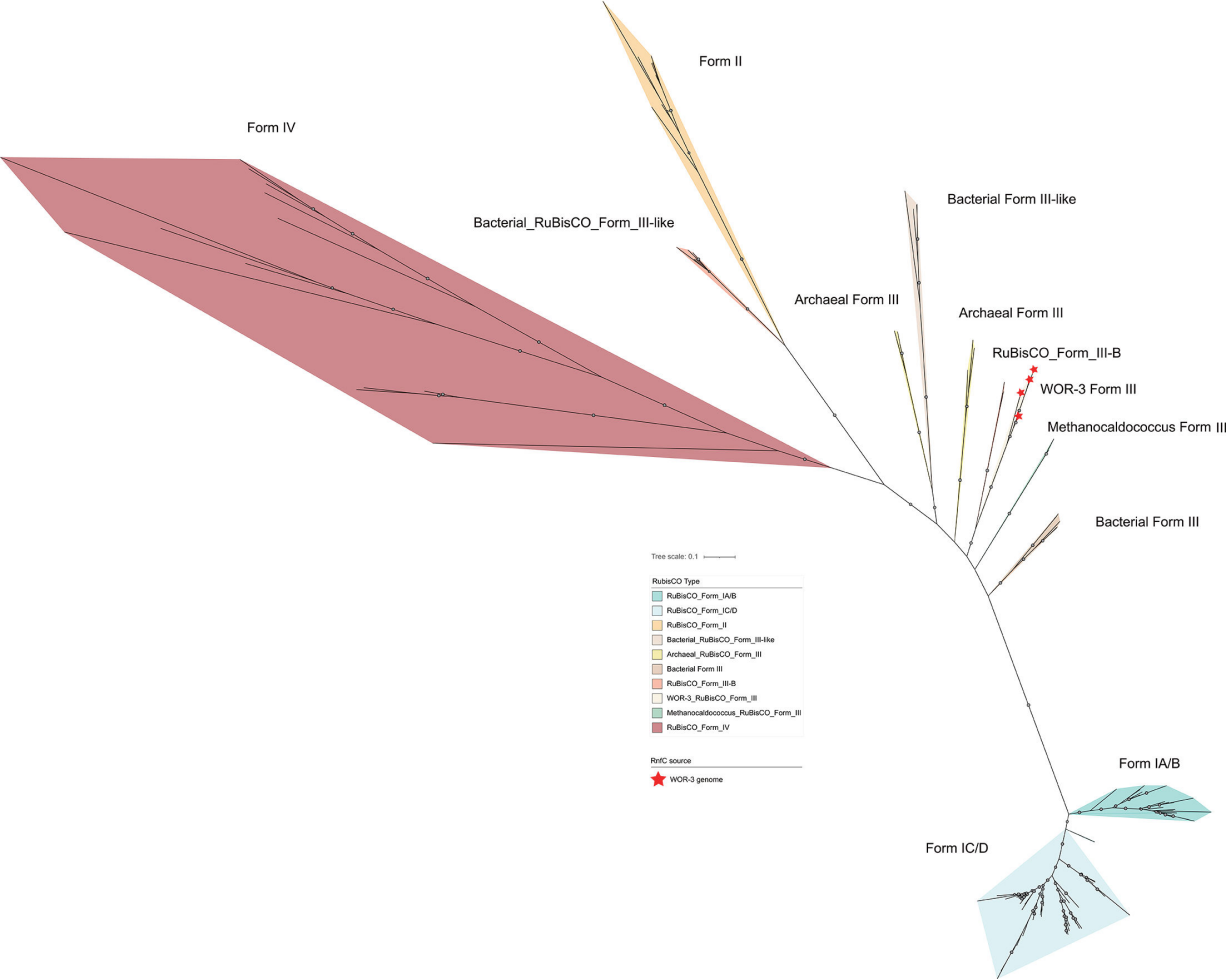
Phylogenetic tree constructed from the RuBisCO protein. RuBisCO forms 10 branches in the phylogenetic tree, and the outgroup branch is form IV type. Red stars indicate RuBisCO sequences from the WOR-3 lineage identified in this study.

This study reveals a previously unreported potential for carbon fixation in the WOR-3 lineage. Metabolic reconstruction analyses have revealed that a proportion of the WOR-3 genome possesses carbon fixation potential, thereby challenging previous assumptions concerning the metabolic capacity of WOR-3. Specifically, five genomes from the genus B3-TA06 under subgroup 1 encode key genes in the carbon fixation pathway, including RuBisCO encoded by the rbcL/S gene and phosphoribulose kinase encoded by the *prk* gene (Fig. 3; Table S4). It is noteworthy that the analysis of the RuBisCO protein sequences using a phylogenetic approach demonstrated that the RuBisCO from WOR-3 is evolutionarily closer to the archaeal RuBisCO form III (Fig. 4). The classification of form III RuBisCO can be divided into two distinct groups: typical form III and form III B (i.e., form II/III). The typical form III is mainly from archaea, and the latter is structurally similar to the bacterial form II but functionally similar to the archaeal form III. Phylogenetic analyses showed that RuBisCO of WOR-3 is closer to form III B (39).

Archaeal RuBisCO form III has three pathways to conduct the Calvin cycle. The first is represented by *Thermococcus kodakarensis* through the nucleoside pathway via the key enzymes AMP phosphorylase (EC2.4.2.57) and omerase (EC5.3.1.29) (39). This pathway dephosphorylates nucleotides (AMP, CMP, UMP), releasing bases to produce ribulose-1,5-bisphosphate (RuBP) and converts to RuBP by ribulose-1,5-bisphosphate isomerase (i.e., AMP → ribulose 1,5-bisphosphate → RuBP). Finally, it converts to two molecules of 3PGA from RuBP and CO_2_ via the form III or form II/III archaeal RuBisCO, participating in central carbon metabolism (40). WOR-3 is unable to metabolize nucleosides via this archaeal pathway due to the absence of the key enzymes AMP phosphorylase (EC2.4.2.57) and isomerase (EC5.3.1.29). The second pathway is abiotic dephosphorylation to ribulose 1,5-bisphosphate, facilitated by archaea (*Methanocaldococcus jannaschii*, *Methanosarcina acetivorans,* and *Archaeoglobus lithotrophicus*) under high temperature conditions (41). In contrast, the WOR-3 MAGs with carbon fixation potential were derived from marine, hypersaline environments, freshwater, and anaerobic bioreactors, respectively, which do not share high temperatures as a commonality. Considering that WOR-3 possesses key genes for the bacterial Calvin cycle, it is hypothesized that carbon fixation in WOR-3 still relies on the bacterial pathway. This phenomenon is not exclusive to WOR-3, but is also exhibited by *Ammonifex degensii* and *Thermodesulfitimonas autotrophica*, which belong to the phylum *Firmicutes* (42). The RuBisCO of WOR-3 is more deeply placed in the phylogenetic tree (Fig. 4). Besides, key genes involved in carbon fixation within the reverse TCA cycle (rTCA cycle) have been identified in 11 genomes from subgroup 3, encompassing citrate synthase (*gltA*), fumarate reductase (*frd*ABCD), and 2-oxoglutarate/2-oxoacid ferredoxin oxidoreductase (*kor*ABCD) (Fig. 3; Table S4). Nevertheless, it should be noted that false-positive results cannot be ruled out on the basis of these data due to the lack of further experimental validation. It is submitted that the carbon fixation potential of WOR-3 extends the knowledge of its metabolic diversity and, moreover, offers novel perspectives for understanding its adaptations in complex environments. This finding highlights the potential importance of WOR-3 in carbon fixation and accordingly lays the foundation for further studies of its ecological functions.

### Energy conservation

The energy metabolic pathways of microorganisms can be classified into two main categories: aerobic and anaerobic metabolism. In such environments, the prevalence and critical importance of nitrogen, sulfur, and methane metabolism is evident, with these pathways involving a variety of electron acceptors such as nitrate, fumarate, arsenate, selenate, thiosulfate, sulfur, sulfate, oxidized metal ions, and carbon dioxide (43, 44).

Significant respiratory divergence within the WOR-3 phylum is evidenced by our identification of two distinct energy-conserving mechanisms: a cytochrome c-dependent respiratory chain enabling oxidative phosphorylation—detected exclusively in 16 MAGs from subgroup 3—and the ubiquitous, sodium-translocating (Na^+^) Rnf complex that functions as the primary respiratory enzyme across the entire phylum, revealing evolutionarily partitioned energy conservation strategies among its subgroup (45).

The oxidative respiratory chain consists of multiple complexes, including complex I (NADH dehydrogenase) encoded by the *nuo* gene, complex II (succinate dehydrogenase flavoprotein) encoded by *sdh*ABCD, complex III (coenzyme Q: cytochrome C oxidoreductase) encoded by *pet*B, and complex IV (cytochrome C oxidase) encoded by *cox*ABC and *cyd*AB. The pure-cultured strain sy37 demonstrated capacity for aerobic/anaerobic respiration using oxygen and elemental sulfur through phenotypic growth assays (4). Genomic analysis revealed four respiratory chain complexes in strain sy37 except for the absence of complex III (Fig. 1; Table S4). This finding challenges the conventional requirement for all respiratory complexes to be intact when determining aerobic/anaerobic lifestyles, thereby reducing the essentiality of complex III—a conclusion consistent with sy37’s criteria of respiratory complexes. Notably, most WOR-3 MAGs lacked all three respiratory complexes (excluding complex III), with only five subgroup 3-affiliated MAGs possessing complete chains. The analysis further revealed that the majority of WOR-3 genomes encode V/A-type ATPases to produce ATP, while a comparatively smaller percentage of genomes possess oxidative enzymes, suggesting a correlation with the predominance of WOR-3 in various environments.

Similarly, the Rnf complex has been found to be widely distributed across all subgroups (135/181 MAGs). The Rnf complex is widely distributed across aerobic, facultatively aerobic, and anaerobic microorganisms but exhibits a distinct preference for anaerobic lifestyles (e.g., *Thermotoga maritima*, *Acetobacterium woodii*), with only a limited number of strict aerobes possessing *rnf* genes (44–48). Phylogenetic analysis of the *rnf* C subunit (Fig. S3) revealed its clustering with anaerobic organisms, thereby further substantiating the anaerobic lifestyle of WOR-3.

**Fig 5:**
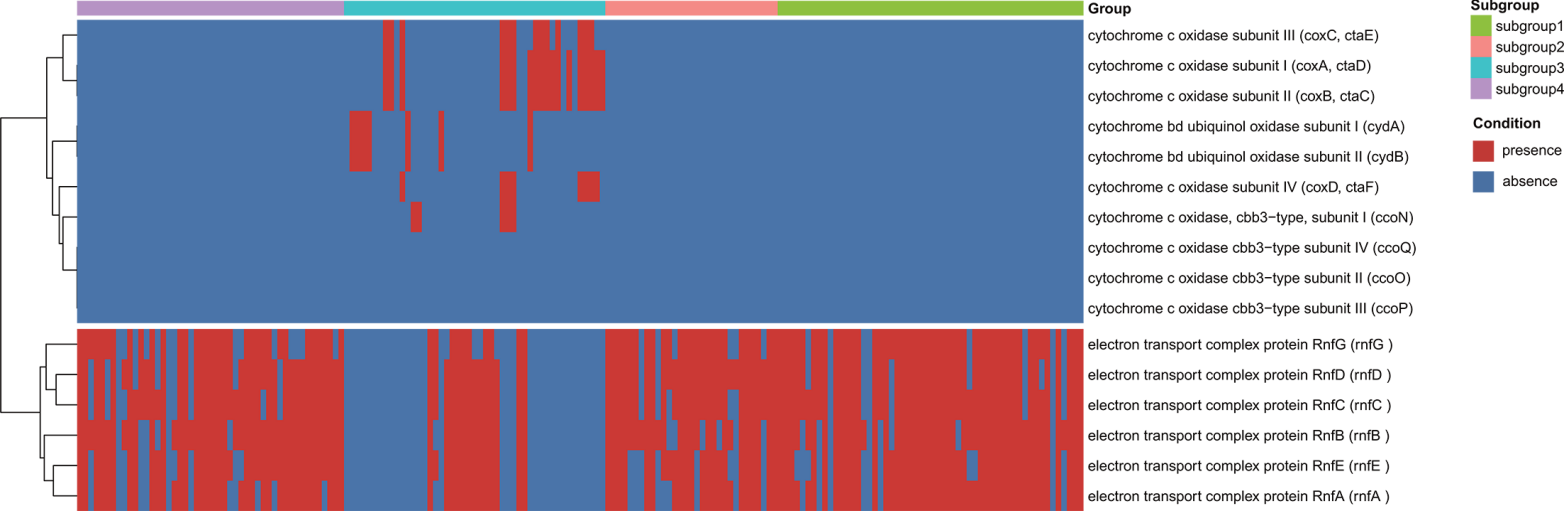
Presence-absence pattern of Rnf complex and cytochrome oxidase subunit genes across the WOR-3 lineage. The heatmap displays a binary matrix, with presence indicated by red and absence by dark blue. Genes (rows) are clustered based on similarity in their presence-absence profiles, and gene names are labeled on the right. Genomes (columns) are ordered by subgroups, with four distinct subgroups highlighted using color bars above the heatmap.

Intriguingly, we observed a mutually exclusive distribution between cytochrome oxidase (indicative of aerobic respiration) and the Rnf complex (associated with anaerobic metabolism), with no MAGs possessing both functional modules (Fig. 5). Consistent with this pattern, genomic analysis of the microaerophilic strain sy37 revealed no *rnf* genes. Combined with the widespread presence of anaerobic fermentation pathways in carbon metabolism and the phylogenetic clustering of RnfC subunits within anaerobic clades, we hypothesize two distinct lifestyle strategies among WOR-3 lineages: (i) an Rnf-dependent anaerobic fermentation mode, and (ii) an aerobic respiratory strategy employing oxygen as the terminal electron acceptor for ATP production via oxidative phosphorylation.

Notably, within subgroup 3, 11 additional MAGs encode complex IV (aa₃-type oxidase) while lacking the Rnf complex, suggesting these populations may also adopt an aerobic lifestyle. Beyond these MAGs, nearly all WOR-3 genomes encode complex I (*nuo* genes) and complex II (*sdh*ABCD). Among auxiliary respiratory components, four MAGs possess *cyd*AB (encoding cytochrome bd ubiquinol oxidase), while 15 MAGs contain *cox*ABC (encoding aa₃-type cytochrome c oxidase) (Fig. 3; Table S4).

### Sulfur metabolism

Regarding the sulfur metabolic capabilities of the WOR-3 lineage, there have been early indications. Phenotypic growth tests of strain sy37 confirm its capacity to utilize elemental sulfur as an electron acceptor. However, canonical sulfur/sulfate reduction genes (*dsr*, *psr*) are notably absent in representative genomes (4). This suggests a potential mechanism analogous to *Pyrococcus furiosus,* which employs SudA-encoded sulfide dehydrogenase-like proteins for sulfur reduction (49). To further explore the sulfur metabolism potential of WOR-3, we analyzed its electron receptor utilization capacity in relation to sulfur metabolism. Sulfhydrogenase (*hyd*ABDG), an enzyme that can transfer polysulfide_*n*_ and H_2_ into polysulfide_(*n*-1)_, H_2_S, and NADH, was found in 42 WOR-3 MAGs (50). The corresponding functions are attributed to subgroups 1, 3, and 4. Furthermore, the results show that 11 MAGs in subgroup 3 support assimilatory sulfate reduction (ASR), with the vast majority (*n* = 10) coming from hydrothermal vent environments and only one from a hot spring environment. Key enzymes involved in ASR include sulfate adenylyltransferase (*sat*), adenylylsulfate kinase (*cys*C), and phosphoadenosine phosphosulfate reductase (*cys*H) (51). The enzymes are distributed in other subgroups, but it is only MAGs from subgroup 3 that possess all three enzymes simultaneously and are able to reduce sulfate to sulfite. However, WOR-3 is unable to further reduce sulfite to sulfide because it lacks sulfite reductase (*cys*JI/*sir*). With regard to the fate of sulfide, it was observed that some MAGs (*n* = 39) possessed cysteine synthase (*cys*K) in various subgroups. These genomes lacked ASR capability, and thus could only synthesize cysteine using exogenous sulfide (Fig. 3; Table S4). Conclusively, WOR-3 could transfer electrons via sulfur metabolism through ASR pathway and sulfur hydrolases that can provide some reducing power.

### Nitrogen metabolism

The nitrogen metabolism in subgroup 3 of WOR-3 is also impressive. DNR, which reduces nitrate to nitrite by nitrate reductase (*nar*GHI), was found in six genomes of subgroup 3. Subgroup 3 also exhibited 10 MAGs containing *nos*Z genes, which encode nitrous-oxide reductase, suggesting its capacity for denitrification through the reduction of nitric oxide to nitrogen gas. WOR-3 genomes with the above-mentioned nitrogen metabolism capacity were found exclusively in subgroup 3 (Fig. 3; Table S4). These observations suggest that subgroup 3 possesses a distinct metabolic profile within the WOR-3 clade, which may be instrumental in fulfilling its ecological function. This disparity in metabolic capacity underscores the diversification among the different subgroups of WOR-3 with respect to ecological adaptation and functional diversity.

### Hydrogen metabolism

In the MAGs of WOR-3, a variety of hydrogenase genes were identified, thus indicating that WOR-3 possesses considerable potential for H₂ metabolism. [NiFe] methyl-viologen-reducing hydrogenase, heterodisulfide reductase, and rnf complex are the most significant due to their prevalence within the WOR-3 metagenome. Specifically, 34 MAGs from various subgroups of WOR-3 possessed both [NiFe] methyl-viologen-reducing hydrogenase (*mvh*ADG) and heterodisulfide reductase (*hdr*ABC), forming a functional complex capable of simultaneous reduction of Fe-oxo-protein and CoB-CoM heterodisulfide during H_2_ oxidation. It is important to note that analogous metabolic mechanisms have been reported in hydrogenotrophic methanogens (52), as well as in certain bacteria such as *Deltaproteobacteria* and *Sumerlaeota* (53, 54). MvhADG-hdrABC2 complex in WOR-3 may be analogous to that in *Sumerlaeota* and involved in energy-efficient metabolism in the population.

### Conclusion

This study provides a comprehensive investigation of the metabolic potential and ecological functions of the phylum WOR-3. Phylogenetic analyses based on 16S rRNA genes and MAGs revealed the global distribution of WOR-3 and its presence across diverse habitats, particularly in aquatic environments such as hot springs, hydrothermal vents, marine, and freshwater systems. The four subgroups of WOR-3 share certain metabolic characteristics, including (i) an anaerobic or aerobic lifestyle; (ii) the potential for H_2_ metabolism; and (iii) carbon metabolism via fermentation and non-oxidative phase of the pentose phosphate pathway. The phylum WOR-3 exhibits substantial diversity in carbon metabolism and ecological functional specialization. Comparative genomic analysis reveals clear distinctions among its four subgroups in core metabolic pathways. Subgroup 1 lacks the genetic potential for glycolysis, gluconeogenesis, and the TCA cycle, indicating an inability to utilize glucose as a primary carbon source. However, it can metabolize alternative substrates via auxiliary pathways such as the xylose isomerase route. In contrast, subgroups 2 and 4 possess complete gene sets for glycolysis, gluconeogenesis, and the TCA cycle, supporting efficient glucose utilization. Subgroup 3 encodes the glycolytic and TCA cycle pathways but lacks gluconeogenic capacity. Notably, this subgroup displays distinct capabilities in sulfur and nitrogen metabolism, including genes associated with ASR, DNR, and denitrification. Additionally, four genomes within subgroup 3 harbor a complete oxidative phosphorylation chain, potentially representing the only members within WOR-3 capable of ATP production via aerobic respiration. Carbon fixation potential is restricted to subgroups 1 and 3, mediated through the CBB cycle and the rTCA cycle, respectively, reflecting divergent ecological strategies across the lineage. Phylogenetic analysis further revealed that WOR-3 encodes a form III archaeal-like RuBisCO, yet its carbon fixation pathway is functionally aligned with the bacterial CBB cycle rather than the archaeal nucleotide-based pathway. This finding provides new insights into the evolution of autotrophic carbon fixation pathways. The metabolic diversity of WOR-3 reflects distinct ecological adaptation strategies. This study not only expands the genomic repertoire of WOR-3 but also offers novel insights into its metabolic capacities and ecological roles, laying a foundation for future investigations into the evolutionary history and ecological significance of uncultivated microbial lineages. Further studies may explore the functional roles of WOR-3 across distinct ecosystems and their interactions with co-occurring microbial taxa, contributing to a more comprehensive understanding of microbial processes in natural environments.

## Data Availability

The fifty-nine genomes retrieved in this study have been deposited at the CNGB Sequence Archive (CNSA) with accession number CNP0007392 and the European Nucleotide Archive (ENA) with accession number PRJEB93962.

## SUPPLEMENTAL MATERIAL

Fig. S1 msystems.01178-25-s0001.pdf ANI-AAI matrix heatmap of WOR-3.

Fig. S2 msystems.01178-25-s0002.eps Phylogenetic tree for 16S rRNA gene.

Fig. S3 msystems.01178-25-s0003.eps Phylogenetic tree constructed based on RnfC subunits from anaerobes as well as aerobes and facultative anaerobes.

Supplemental text msystems.01178-25-s0004.docx Supplemental legends.

Supplemental tables msystems.01178-25-s0005.xlsx Tables S1-S4.

ASM does not own the copyrights to Supplemental Material that may be linked to, or accessed through, an article. The authors have granted ASM a non-exclusive, world-wide license to publish the Supplemental Material files. Please contact the corresponding author directly for reuse.
