# Supplementary figures and images for: Genome-resolved insights into the bacterial phylum WOR-3: hydrogenotrophic metabolism and unique carbon fixation via archaeal form III RuBisCO
Source: mSystems. 2025 Oct 2;10(10):e01178-25. doi: 10.1128/msystems.01178-25 (PMC12542636; doi:10.1128/msystems.01178-25)

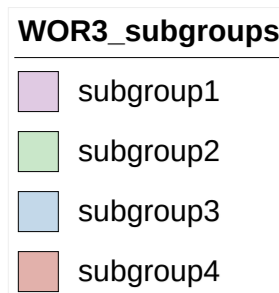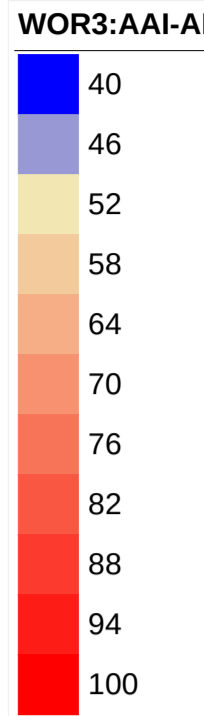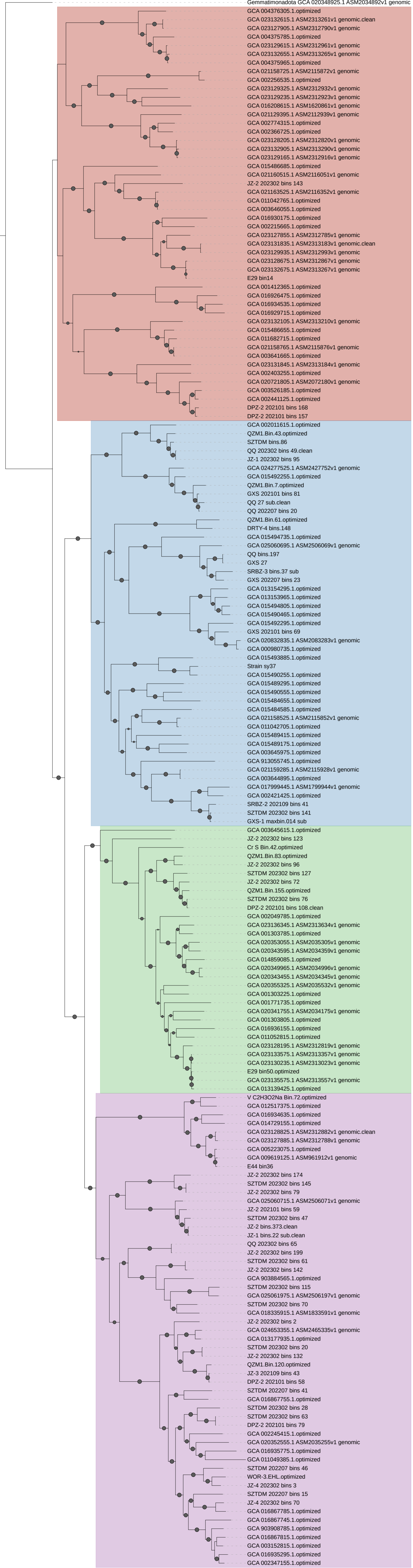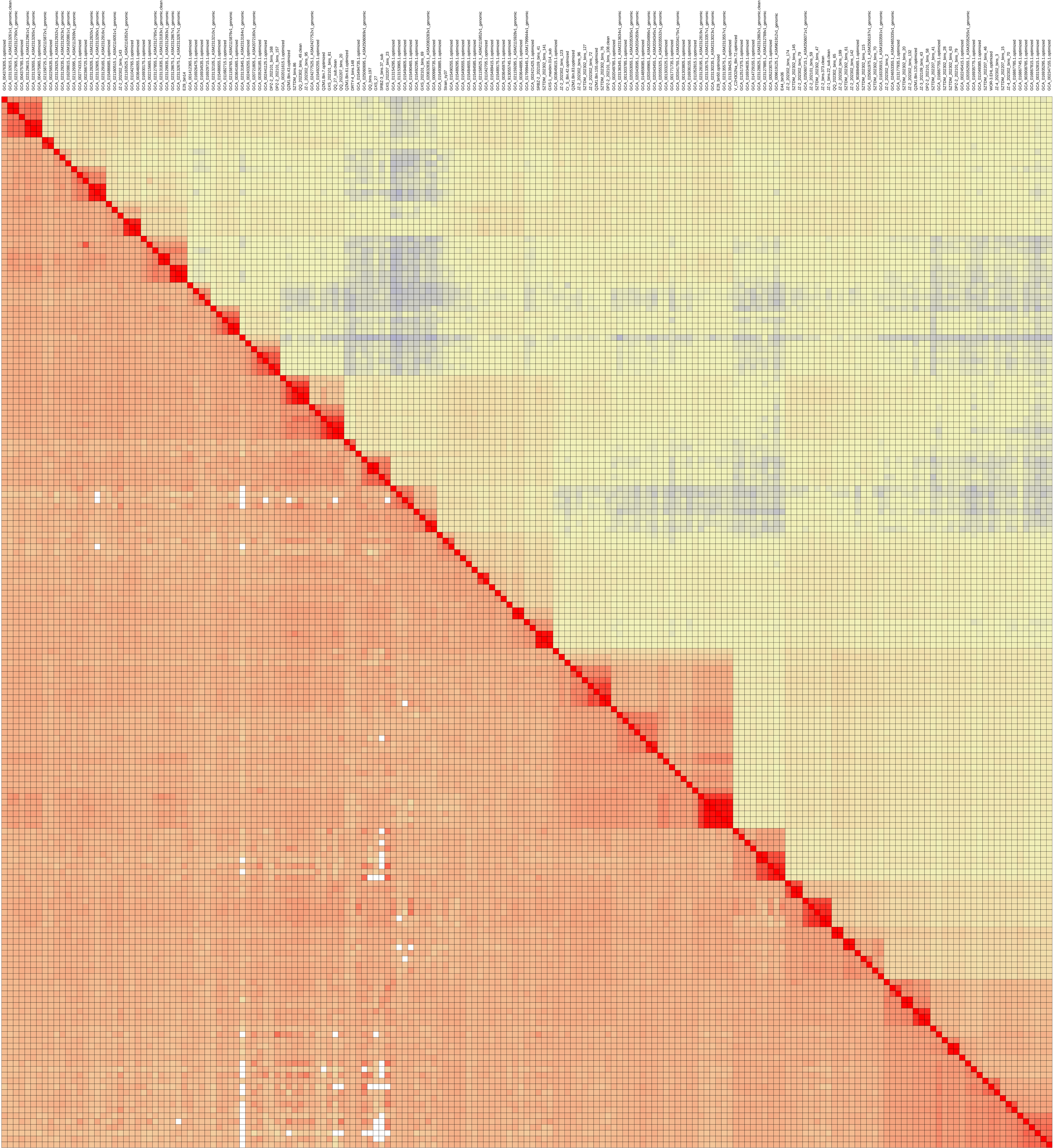

Supplement: Fig. S1 — ANI-AAI matrix heatmap of WOR-3. [file msystems.01178-25-s0001.pdf]
