# Supplementary material for: Genome-resolved insights into the bacterial phylum WOR-3: hydrogenotrophic metabolism and unique carbon fixation via archaeal form III RuBisCO
Source: mSystems. 2025 Oct 2;10(10):e01178-25. doi: 10.1128/msystems.01178-25 (PMC12542636; doi:10.1128/msystems.01178-25)
Supplement: Supplemental text — Supplemental legends. [file msystems.01178-25-s0004.docx]

**Supplemental legends**

**Fig. S1** - ANI-AAI matrix heatmap of WOR-3. The reliability of the phylogenetic tree is supported by the ANI-AAI matrix heatmap, and the white squares in the heatmap indicate outliers calculated by ANI.

**Fig. S2** - Phylogenetic tree for 16S rRNA gene. Different classes are indicated by distinct color ranges along the outer edge of the tree. Bootstrap values greater than 75% are highlighted with solid grey rectangles bordered in black, placed at the corresponding nodes.

**Fig. S3** - Phylogenetic tree constructed based on RnfC subunits from Anaerobes as well as Aerobes and facultative anaerobes. Bootstrap values for phylogenetic trees greater than 75 are marked with grey solid black-edged circles.

**Table S1** Genomic characteristics of WOR-3 MAGs.

**Table S2** Functional annotation and taxonomic classification summary of WOR-3 MAGs.

**Table S3** WOR-3 16S rRNA sequences traceability information.

**Table S4** Metabolic pathway gene annotations in WOR-3 MAGs.
